# A Rhodococcal Transcriptional Regulatory Mechanism Detects the Common Lactone Ring of AHL Quorum-Sensing Signals and Triggers the Quorum-Quenching Response

**DOI:** 10.3389/fmicb.2018.02800

**Published:** 2018-11-19

**Authors:** Corinne Barbey, Andrea Chane, Jean-François Burini, Olivier Maillot, Annabelle Merieau, Mathias Gallique, Amélie Beury-Cirou, Yoan Konto-Ghiorghi, Marc Feuilloley, Virginie Gobert, Xavier Latour

**Affiliations:** ^1^Laboratoire de Microbiologie Signaux et Microenvironnement (LMSM EA 4312) – Normandie Université – LMSM, Évreux, France; ^2^Structure Fédérative de Recherche Normandie Végétal 4277 (NORVEGE), Mont-Saint-Aignan, France; ^3^Seeds Innovation Protection Research and Environment, Achicourt, France; ^4^Seeds Innovation Protection Research and Environment, Bretteville du Grand-Caux, France; ^5^French Federation of Seed Potato Growers (FN3PT/RD3PT), Paris, France

**Keywords:** biocontrol, Actinobacterium, *Rhodococcus*, TetR-like regulator, metabolism

## Abstract

The biocontrol agent *Rhodococcus erythropolis* disrupts virulence of plant and human Gram-negative pathogens by catabolizing their *N*-acyl-homoserine lactones. This quorum-quenching activity requires the expression of the *qsd* (quorum-sensing signal degradation) operon, which encodes the lactonase QsdA and the fatty acyl-CoA ligase QsdC, involved in the catabolism of lactone ring and acyl chain moieties of signaling molecules, respectively. Here, we demonstrate the regulation of *qsd* operon expression by a TetR-like family repressor, QsdR. This repression was lifted by adding the pathogen quorum signal or by deleting the *qsdR* gene, resulting in enhanced lactone degrading activity. Using interactomic approaches and transcriptional fusion strategy, the *qsd* operon derepression was elucidated: it is operated by the binding of the common part of signaling molecules, the homoserine lactone ring, to the effector-receiving domain of QsdR, preventing a physical binding of QsdR to the *qsd* promoter region. To our knowledge, this is the first evidence revealing quorum signals as inducers of the suitable quorum-quenching pathway, confirming this TetR-like protein as a lactone sensor. This regulatory mechanism designates the *qsd* operon as encoding a global disrupting pathway for degrading a wide range of signal substrates, allowing a broad spectrum anti-virulence activity mediated by the rhodococcal biocontrol agent. Understanding the regulation mechanisms of *qsd* operon expression led also to the development of biosensors useful to monitor *in situ* the presence of exogenous signals and quorum-quenching activity.

## Introduction

Bacteria use cell-to-cell communication systems based on both synthesis and perception of signaling molecules to synchronize their social behavior. These quorum-sensing (QS) systems control diverse functions, which require the concerted actions of numerous cells in order to be productive, such as antibiotic synthesis, motility, symbiosis, biofilm maturation, sporulation, and virulence (Papenfort and Bassler, 2016). Among them, the most-studied system relies on the production of molecules belonging to the family of *N*-acyl-L-homoserine lactones (AHLs). These play a key role in the pathogenicity of a dozen of clinically relevant pathogens and many agronomically important pathogens (von Bodman et al., 2003; Rutherford and Bassler, 2012). For example, *Pseudomonas aeruginosa*, one of the leading opportunistic pathogens, uses several QS networks to control the production and release of multiple virulence factors such as LasA and LasB elastases, exotoxin A and alkaline protease involved in acute pulmonary infection (Jimenez et al., 2012). A mutant deficient in the synthesis of AHL signal molecules was nearly avirulent in a murine model of acute pulmonary infection (Pearson et al., 2000). Among the top 10 phytopathogens, *Pectobacterium* spp. employs QS that can also harbor a high degree of complexity to integrate several AHL signaling networks (Barnard and Salmond, 2007; Mansfield et al., 2012; Valente et al., 2017). Pectobacterial QS ensures the synchronized production of massive amounts of lytic enzymes involved in plant tissues degradation (Barnard and Salmond, 2007; Põllumaa et al., 2012). Many AHL-controlled functions have already been reported in the *Pectobacterium atrosepticum* species. They comprise up to a quarter of its genome, in particular a huge arsenal of plant cell-wall degrading enzymes as well as their secretion systems (Liu et al., 2008). Therefore, a single mutation of the AHL synthase gene, AHL receptor/transcription factor, or the degradation of signal molecules before their release in the microenvironment is sufficient to remove all symptoms on host plants (Smadja et al., 2004b; Latour et al., 2007; Põllumaa et al., 2012).

Since QS is essential for disease progression in many pathogenic bacteria, disrupting the microbial QS system by a quorum-quenching (QQ) mechanism appears as a promising anti-virulence strategy for human and plant health (Dong et al., 2001, 2007; Njoroge and Sperandio, 2009; LaSarre and Federle, 2013; Helman and Chernin, 2015; Dessaux et al., 2016). Indeed, QQ aims at reducing virulence expression rather than limiting cell growth or eradicating pathogens. It may involve the use of bacteria as well as their products to overcome the risk of microbial invasion, a role and applications exhaustively described in the recent reviews of Grandclément et al. (2016) and Zhang and Li (2016). Furthermore, this QQ strategy is considered more sustainable of the microbial balance (Bais, 2012). If it involves hydrolysis of signals, it also limits the development of resistance because of a less selective pressure on bacterial populations than antibiotics or QS inhibitor molecules (Defoirdt et al., 2010; Mellbye and Schuster, 2011; Garcia-Contreras et al., 2013; Garcia-Contreras, 2016; Guendouze, 2017).

The first reports of QQ activities were carried out in soilborne bacteria belonging to *Bacillus* or *Variovorax* genera (Dong et al., 2000; Leadbetter and Greenberg, 2000), then joined by other quencher models associated with plants such as *Agrobacterium tumefaciens* or *Rhodococcus erythropolis* (Zhang et al., 1993; Uroz et al., 2003). In the rhodococcal model, enzymes that catabolize AHLs are mainly lactonases that open the homoserine lactone ring (Uroz et al., 2003; Oh et al., 2013) or amidases (also referred as acylases or amidohydrolases) that cleave AHL amide bond and release the homoserine lactone (HSL) and a fatty acid (Lin et al., 2003; Uroz et al., 2005). We screened and isolated the *R. erythropolis* strain R138, a biocontrol bacterium able to degrade effectively diverse AHL signals (Afriat et al., 2006; Cirou et al., 2007; Oh et al., 2013) and to suppress the disease in hydroponic and field culture conditions (Cirou et al., 2011, 2012). The molecular mechanism of this protection was demonstrated *in vivo* and mainly involves a new pathway (Barbey et al., 2012, 2013). This pathway requires the expression of the *quorum-sensing signal degradation* (*qsd*) operon encoding the phosphotriesterase-like lactonase QsdA (EC 3.1.1.81) for the lactone bond hydrolysis, and the fatty acyl-CoA ligase QsdC (syn. FadD, EC 6.2.1.3) for activation of acyl chains before their oxidation or recycling (Uroz et al., 2008; Latour et al., 2013). The latter was named QsdC because QsdB was previously proposed for a QS-signal degrading amidase (El Sahili et al., 2015). Besides, an AHL amidolytic activity was initially identified by HPLC analysis and chemical trapping of HSL in a *R. erythropolis* W2 crude cell extract (Uroz et al., 2005). Then, proteomic analysis enabled to identify an amidase (NCBI reference WP_019744590.1) produced by *R. erythropolis* R138 during growth only in the presence of AHL as sole carbon source (Latour et al., 2013). Interestingly, the rhodococcal biocontrol activity is induced not by the invasion step or the massive presence of the pathogen, but by its QS-based communication; indeed, no *qsd* operon transcription was observed in the presence of pathogens defective for AHL release (Barbey et al., 2013). This AHL induction phenomenon suggests the existence of a regulatory system for the *qsd* operon transcription. Sequence analysis of the upstream region of the *qsd* operon revealed a putative TetR family transcriptional regulator gene, that we named *qsdR* (Latour et al., 2013), and which belongs to a transcriptional repressor family (Ramos et al., 2005; Cuthbertson and Nodwell, 2013). Then, a substantial biochemical analysis enabled to define the crystal structure of QsdR protein and a genome engineering approach exhibited that the change of only one amino-acid in QsdR leads to enhancement of the QS-signal degradation by *R. erythropolis* (El Sahili et al., 2015). We describe here the role of QsdR and AHL molecules in the regulation of the *qsd* operon transcription and propose a model in which QQ activity is triggered by various AHL signals.

## Materials and Methods

### Bacterial Strains, Growth, and Culture Conditions

The characteristics of bacterial strains and plasmids are presented in Supplementary Table S1. *R. erythropolis* strains were cultivated in LBP (Barbey et al., 2013) for conjugative transfer of DNA and in 7H9 minimal medium (Difco) supplemented with hexanoate at 6 mM (Sigma-Aldrich) as carbon source for induction assays. Induction experiments were performed by adding at the mid-exponential phase the *N*-3-oxo-octanoyl-L-homoserine lactone (3 oxo-C_8_-HSL) (Sigma-Aldrich) molecules at concentrations ranging from 10 nM to 3 mM. Luria-Bertani medium (AES Chemunex, Bruz, France) was used for the cultivation of *E. coli* strains. Pathogens, *P. atrosepticum* 6276 and *P. aeruginosa* PA14, were grown in PGA minimal medium supplemented with 0.4% (w/v) polygalacturonic acid (Sigma-Aldrich, St. Louis, United States) as described elsewhere (Barbey et al., 2013), and King B medium, respectively. When necessary, growth media were supplemented with kanamycin at a concentration of 30 μg/ml for *E. coli* and 200 μg/ml for *R. erythropolis*, with apramycin at 50 μg/ml for *E. coli* and 80 μg/ml for *R. erythropolis*, with ampicillin at 100 μg/ml for *E. coli* and solidified with agar (15 g/l). All bacteria were grown on a rotary shaker (180 rpm) at 25°C for *R. erythropolis* strains and *P. atrosepticum* 6276 and 37°C for *E. coli* and *P. aeruginosa* PA14.

### Construction and Complementation of Δ*qsdR* Mutant in *R. erythropolis* Strain R138

A markerless *qsdR* deletion mutant (Δ*qsdR*) was constructed as previously described (Barbey et al., 2013) using the oligonucleotide pairs qsdR_up_fw/qsdR_up_rv and qsdR_do_fw/qsdR_do_rv (Supplementary Table S2) for the amplification of the *qsdR* upstream and downstream regions (881 and 878 bp), respectively. The in-frame *qsdR* deletion mutant containing a truncated version of the *qsdR* gene (69 bp in length instead of 558 bp for the wild-type gene) was verified by PCR analysis and DNA sequencing.

To generate the complemented strain, the *qsdR* gene with its promoter was amplified with the Extensor Hi Fidelity polymerase using primers pSET152_qsdR_F and pSET152_qsdR_R (Supplementary Table S2) and cloned as an 855-bp EcoRI-XbaI fragment into the integrative vector pSET152 (Swain et al., 2012). After sequencing of the insert, this plasmid designated pSET152-*qsdR* was introduced in *R. erythropolis* R138 cells by biparental mating as previously described (Barbey et al., 2013). The complemented strain was called Δ*qsdR-qsdR*.

### Biocontrol Assays on Potato Tubers

*Solanum tuberosum* cv. Allians tubers were prepared for inoculation as previously described (Barbey et al., 2013). They were inoculated by injection into the intramedulla (to a depth of 1 cm) with 10 μl of cell suspension containing the following bacterial combinations: [10^7^ CFU of *P. atrosepticum* CFBP6276 with 10^8^ CFU of *R. erythropolis* R138/R138 Δ*qsdA/E. coli* DH5α(pUC19)/DH5α(pUC19-*qsdA*)] or [10^8^ CFU of *P. aeruginosa* PA14 with 10^8^ CFU of *R. erythropolis* R138/R138 Δ*qsdA*/*E. coli* DH5α(pUC19)/DH5α(pUC19-*qsdA*)]. For controls, the suspensions of *P. atrosepticum* CFBP6276, *P. aeruginosa* PA14 and/or *R. erythropolis* R138 were replaced with 0.9% NaCl. The *P. atrosepticum* and *P. aeruginosa* inoculated tubers were incubated in a Minitron incubator (Infors, Massy, France) with a relative humidity of 80% at 25 and 30°C, respectively. At 7 days after inoculation, 30 tubers for each condition were sectioned across the middle, photographed, analyzed by measuring maceration diameter and cut for bacterial population analysis as previously described (Barbey et al., 2013). These experiments were conducted three times. The Mann and Whitney test was used to assess differences in maceration symptoms between groups (*p*-value < 0.01).

### Colorimetric Quantification of Quorum Signals

The 3-oxo-C_8_-HSL molecules used for induction assays were quantified by a colorimetric method typically applied to the analysis of ester molecules (Yang et al., 2006). This method is based on the formation of hydroxamic acids from esters by reaction with hydroxylamine in alkaline solution. The hydroxamic acid forms a highly colored complex with ferric ion, which is spectrophotometrically measurable at 520 nm. Dosage was performed as described by Cirou et al. (2012) with the following modifications. Culture supernatants were collected every 2 h and centrifuged at 5,000 *g* for 5 min. Stability of 3-oxo-C_8_-HSL in 7H9 medium was checked by incubating a non-inoculated 7H9 medium under the same conditions as inoculated media. A volume of 200 μl of supernatant or the non-inoculated medium was successively mixed with 250 μl of 2 M hydroxylamine (Sigma-Aldrich)/3.5 M NaOH (Merck) (1:1, v/v) and 250 μl of 10% iron chloride (Sigma-Aldrich) prepared in 4 M HCl / 95% Ethanol (Merck) (1:1, v/v). The absorbance of the mixture was measured at 520 nm against a blank consisting of the same mixture in which culture supernatant was replaced with the non-inoculated 7H9 medium without 3-oxo-C_8_-HSL.

### Quantitative RT-PCR

RNA was extracted by a modified version of the phenol-based extraction procedure described by Crépin et al. (2012a). Cells were first lysed by vortexing twice for 5 min with acid-washed glass beads in the lysis buffer [0.02 M sodium acetate, pH 5.5, 0.5% (w/v) SDS, 1 mM EDTA]. mRNAs were quantified by real-time PCR as described by Bouffartigues et al. (2015) using primers in the Supplementary Table S2. The relative quantification of the mRNAs was obtained by the comparative CT (2^-ΔΔCT^) method as described (Bouffartigues et al., 2015). This analysis was performed using the recombinase A (*recA*) gene as endogenous control, as previously described for *R. erythropolis* gene expression study (Kwasiborski et al., 2015).

### *In silico* Studies and Molecular Docking

The *R. erythropolis* R138 genome sequence is available from the NCBI database with the accession no. NZ_CM002793.1. The *qsd* cluster is composed of *qsdR* (gene ID H351_RS26320), *qsdA* (gene ID H351_RS26315), and *qsdC* (gene ID H351_RS26310). The *qsdR* gene is located upstream of a FadR encoding gene (gene ID H351_RS05745). The clone manager software (professional edition version 9.00) was used to identify palindrome motifs. Regulator binding sites were predicted using the web-based software bprom in the Softberry package (Solovyev and Salamov, 2011). The crystallized structure of the *R. erythropolis* R138 QsdR (receptor) was obtained from the Protein Data Bank (PDB) with the accession number 4ZA6. The structure was downloaded in a.pdb format and was cleaned by removing co-crystallized ligands and water by using Discovery Studio 4.5, and finally save in.pdb format. The potential ligands were drawn by using Discovery Studio 4.5 and save in.pdb format. The docking was carried out with AutoDock Vina (Trott and Olson, 2010) by using AutoDock Tools 1.5.6 for preparing the receptor and each ligand tested (Morris et al., 2009). Potential ligand–protein interactions were investigated *in silico* by molecular docking. H-atoms of the receptor were added for correct ionization and tautomeric states of amino acid residues with AutoDock Tools 1.5.6 (Morris et al., 2009; Trott and Olson, 2010). Docking simulations were made using the Lamarckian genetic algorithm (LGA). Kollman united atom type charges and solvation parameters were added with AutoDock Tools 1.5.6 (Morris et al., 2009). Initial position, orientation and torsions of the ligands were set randomly. The energy scoring grid box was centered in the middle of the receptor and was set to 126, 126, and 126 Å (x, y, and z) centered at X = -22.709; Y = -9.312 and Z = 10.331 with 0.753 Å spacing. AutoDock Vina generated the docking results (ΔG) in a.pdbqt file in kcal/mol, subsequently transformed in kJ/mol.

### Mapping of Transcription Start Sites

5′ Rapid Amplification of cDNA ends (RACE) was performed to define the transcription start site (TSS) of the operon *qsd* and of the *qsdR* gene using primers listed in the Supplementary Table S2 and the 5′ RACE System, Version 2.0 (Invitrogen) according to the manufacturer’s instructions. PCR products were cloned into pGEM-T Easy vector according to the manufacturer’s instructions (Promega) and sequenced to identify the 5′ end of the *qsdA* and *qsdR* mRNA (Beckman Coulter Genomics, Villepinte, France).

### Electrophoretic Mobility Shift Assay (EMSA)

The *qsdR* ORF was amplified with Extensor Hi Fidelity polymerase using primers pET19-qsdR F and pET19-qsdR R (Supplementary Table S2) and cloned as a 570-bp EcoRI-BamHI fragment into the expression vector pET19. The *E. coli* BL21 (DE3) cells were transformed with the resulting pET19-*qsdR* vector and were grown at 20°C in LB medium containing ampicillin (100 mg/l). When absorbance at 580 nm reached 0.6, isopropyl-1-thio-β-D-galactoside (IPTG) was added at a final concentration of 0.15 mM and the cells were harvested 12 h later by centrifugation. QsdR recombinant protein was purified using Ni NTA agarose as described by the manufacturer (Qiagen). Complementation experiments with the Δ*qsdR* strain were performed to verify that the His tag does not interfere with the QsdR function. The *qsdR qsdA* intergenic DNA fragment used in EMSA was amplified using the qsdR-qsdA-EMSA F and qsdR-qsdA-EMSA R primers (Supplementary Table S2) with the genomic DNA of *R. erythropolis* R138 as template. EMSA was performed with 0.3 to 3 μg of purified His tagged QsdR, and 0.4 ng DNA fragment using LightShift^®^ Chemiluminescent EMSA Kit as described by the manufacturer (Thermo Scientific). For negative control, qsdA-qsdA-EMSA F and qsdA-qsdA-EMSA R primers were used to amplify a biotin labeled DNA internal fragment of *qsdA* between the nucleotide positions 431 and 607 from the start codon. The effect of HSL or AHLs on the ability of QsdR to bind the *qsdR qsdA* intergenic DNA fragment were tested by adding a ligand/QsdR molecules ratio increasing from 0.1 to 100 into the 20 μl EMSA binding reaction. In control samples, ligand solution was replaced by ethyl acetate to check the lack of solvent effect on protein/DNA binding.

### Mass Spectrometry Analysis

Protein spots to be identified were manually excised from Coomassie stained SDS-PAGE. Excised gel fractions were washed in 100 mM ammonium bicarbonate (Sigma-Aldrich)/acetonitrile (Merck) (1:1, v/v) until completely destained. After drying with acetonitrile, gel fragments were rehydrated in 20 μl of trypsin solution (Trypsin Gold, Mass Spectrometry grade, Promega) at 13 ng/μl in 10 mM ammonium bicarbonate solution containing 10% acetonitrile (v/v). Then, digestion was performed at 37°C for 16 h. Peptide extraction was carried out twice for 1 h with 20 μl 50% acetonitrile/5% formic acid (Sigma-Aldrich). In-gel trypsin digestion products were analyzed by a Matrix Assisted Laser Desorption Ionization Time-of-Flight mass spectrometer (MALDI-TOF/TOF LIFT, AutoFlex III, Bruker Daltonics) in positive/reflector mode controlled by FlexControl software Version 3.3, as previously described (Barbey et al., 2012).

### Construction and Analysis of Transcriptional Fusion Strains by Confocal Laser Scanning Microscopy (CLSM)

The pEPR1 promoter probe vector (Knoppová et al., 2007) was modified to construct three derivative vectors. The pEPR1-*mCherry* vector was built by introducing the *mCherry* cassette to constitutively tag rhodococcal cells. The *mCherry* cassette was amplified with the primers mCherry_F and mCherry_R (Supplementary Table S2) from the pPSV35-*mCherry* plasmid DNA and cloned into pEPR1 after *Nhe*I digestion. The pEPR1-P*qsd*::*gfp-mCherry* was made by amplifying the *qsdA* promoter region using primers Pqsd_F and Pqsd_R (Supplementary Table S2). The resulting PCR product was cloned into the pEPR1-*mCherry* vector after digestion by *Nsi*I and *Bam*HI. For the construction of the pEPR1-*qsdR*-P*qsd*::*gfp-mCherry*, the DNA fragment containing the *qsdR* gene and the *qsd* promoter was introduced into the pEPR1-*mCherry* vector after PCR amplification using the primers qsdR-Pqsd_F and qsdR-Pqsd_R (Supplementary Table S2) and digestion by *Nsi*I and *Bam*HI. All PCR, ligation and restriction reactions were carried out with Phusion Hi Fidelity polymerase (Biolabs NEB), T4 DNA ligase (Biolabs NEB) and NEB endonucleases in accordance with the supplier’s recommendations, respectively. Each resulting vector was introduced into the *R. erythropolis* R138 cells by electroporation as previously described (Barbey et al., 2013). Bacterial strains transformed with plasmids harboring reporter genes were cultivated in the same medium and under the same conditions as those used for *qsd* expression assays by qRT-PCR. For induction experiments, different concentrations of HSL or AHLs (1 μM to 1 mM) were added at mid-exponential phase. Analysis of induced and non-induced cultures by confocal microscopy was performed by fixing bacteria with ethanol on glass slides. Slides were examined by inverted CLSM (LSM 710, Carl Zeiss MicroImaging, Le Pecq, France). To excite the GFP or *mCherry* in bacterial cells, a 488 or 594 nm laser with 509 or 610 nm emission filters was used, respectively. Confocal images were acquired with Zen 2009H software (Carl Zeiss MicroImaging) using the same gains and offset parameters for all images. Three bacterial smears from three independent culture conditions were analyzed for each condition.

## Results

### The *qsd* Operon Is the Source of the Anti-virulence Activity of *R. erythropolis* Against AHL-Producing Gram-Negative Pathogens

**FIGURE 1:**
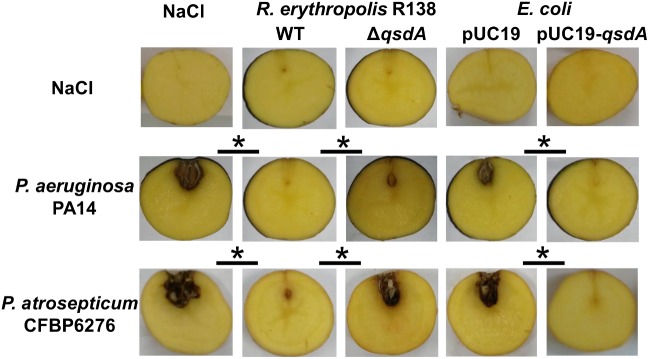
Anti-virulence activity of *Rhodococcus erythropolis* and QsdA lactonase expressing *E. coli* strains against AHL-producing Gram-negative pathogens. Quorum quenchers and their corresponding control strains (*R. erythropolis* Δ*qsdA* mutant strain defective in QsdA lactonase production and *E. coli* pUC19) were compared for biocontrol activity against a 3-oxo-C_12_-HSL and C_4_-HSL producer, the human pathogen *P. aeruginosa* PA14, as well as a 3-oxo-C_8_-HSL producer, the potato pathogen *Pectobacterium atrosepticum* CFBP6276. Seven days after inoculation in potato tubers, tissue necrosis or soft-rot due to *P. aeruginosa* and *P. atrosepticum*, respectively, were analyzed and compared between tuber lots (black bars). For the controls, one or both strains were replaced in the inoculum with a 0.9% NaCl solution. Significant differences (Mann and Whitney test; *p*-value < 0.01) in maceration symptoms between infected tubers inoculated with control and quorum quencher strains are indicated with an asterisk.

*Rhodococcus erythropolis* strain R138 was isolated from the *S. tuberosum* potato rhizosphere in which it degrades effectively AHLs (Cirou et al., 2011; Barbey et al., 2013). Involvement of *qsd* operon in the anti-virulence activity of *R. erythropolis* against the human pathogen *P. aeruginosa* PA14 and the potato pathogen *P. atrosepticum* CFBP6276 was studied using a plant infection model sensitive to both pathogens (Rahme et al., 1997; Smadja et al., 2004a). This potato tuber assay makes it easy to quantify the bacterial virulence by measuring the extent of cellular damage (i.e., maceration) (Crépin et al., 2012b). *P. aeruginosa* is well known to use two AHL-based QS systems regulating the production of many virulence factors: the *las* and *rhl* systems produce and respond to the *N*-3-oxo-dodecanoyl-L-homoserine lactone (3-oxo-C_12_-HSL) and *N*-butanoyl-L-homoserine lactone (C_4_-HSL), respectively (Jimenez et al., 2012; Mund et al., 2017). In contrast, *P. atrosepticum* produces and responds mainly to the *N*-(3-oxo-octanoyl)-L-homoserine lactone (3-oxo-C_8_-HSL) in order to coordinate its attack and overwhelm the host plant defenses (Latour et al., 2007; Crépin et al., 2012c). To investigate the role of the *qsd* operon in biocontrol, anti-virulence activity of *R. erythropolis* R138 was compared with a *R. erythropolis* mutant strain defective in the production of the lactonase QsdA. The key role of QsdA in protection was then validated by its heterologous expression in *Escherichia coli*, a bacterium unable to degrade AHLs (Figure 1). First, the *qsdA* gene was introduced into *E. coli* DH5α, and the heterologous expression of QsdA was verified by SDS PAGE analysis and MALDI-TOF sequencing. For tubers infected with *P. aeruginosa*, drastic differences in tissue maceration were observed between tubers co-inoculated with the quorum quencher strains (*R. erythropolis* R138 and *E. coli* pUC19-*qsdA*) and those co-inoculated with their derivative strains (*R. erythropolis* R138 Δ*qsdA* and *E. coli* pUC19) 7 days after inoculation. The same results were obtained for tubers infected with *P. atrosepticum* (Figure 1). Under all assay conditions, pathogen and antagonist populations grew in mixed populations, with *P. aeruginosa* PA14 and *P. atrosepticum* CFBP6276 populations reaching 10^8^ and 10^9^ CFU/g of fresh tuber after 7 days, respectively, confirming that *R. erythropolis* R138 did not express antibiotic activities against these bacteria as already observed (Barbey et al., 2013; Kwasiborski et al., 2015).

### *qsd* Operon Transcription Is Induced by QS Signals

**FIGURE 2:**
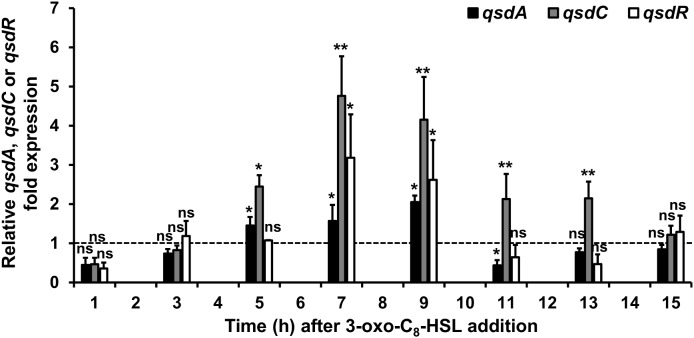
Induction of the *qsd* operon expression by 3-oxo-C_8_-HSL. qRT-PCR analysis of the *qsdA*, *qsdC*, and *qsdR* transcription was conducted in the *R. erythropolis* R138 wild-type strain grown in 7H9 medium in which 3-oxo-C_8_-HSL was added at mid-exponential phase. Expression levels of *qsdA*, *qsdC*, and *qsdR* are relative to those obtained in the same medium without the inducer. The dotted line indicates the same mRNA expression between the induced condition and without the inducers. Data shown are mean values obtained from three independent experiments. Statistical analysis was performed by the DataAssist^TM^ software (v3.01) used for calculating relative quantitation of gene expression, based on the comparative *C*_T_ (2^-ΔΔCT^) method. ^∗^*p*-value < 0.05; ^∗∗^*p*-value < 0.01; ns means no significant difference.

To investigate the induction mechanism of the rhodococcal QQ activity, *R. erythropolis* was grown in a minimal medium containing hexanoate as the sole carbon source. This aliphatic compound was chosen because it is not able to form a lactone ring and its assimilation does not induce the production of lactonase QsdA (Barbey et al., 2012). The 3-oxo-C_8_-HSL was used as a model inducer for its prevalence in numerous plant-associated bacteria (Cha et al., 1998; D’Angelo-Picard et al., 2005) including the *P. atrosepticum* species (Latour et al., 2007; Crépin et al., 2012c). The 3-oxo-C_8_-HSL was added to the *R. erythropolis* R138 culture at mid-exponential growth phase. Bacterial cells were then harvested every 2 h for the *qsd* operon expression analysis by qRT-PCR and 3-oxo-C_8_-HSL degradation kinetics. Expression levels were compared with expression levels obtained from the same culture conditions without the addition of the AHL molecules. Exposure of *R. erythropolis* to 3-oxo-C_8_-HSL induced a significant increase in *qsdA* and *qsdC* expression levels. For both genes, the transcription levels increased significantly from 5 h, reached a maximum between 7 and 9 h after induction and then decreased (Figure 2).

### Identification of a *qsdR* Gene Upstream of the *qsd* Operon: Influence of This Gene on *qsd* Expression and QS Signal Degradation

The complete genome sequence of the biocontrol strain *R. erythropolis* R138 was recently achieved. It consists of a circular chromosome (about 6.7 Mbp) and two plasmids (Kwasiborski et al., 2015). Sequence analysis of the upstream region from the R138 chromosomal *qsd* operon revealed the presence of a gene encoding a putative TetR family transcriptional regulator, QsdR (Supplementary Figure S1). Due to its key position, this gene was already suspected to play a key role in the regulation of the *qsd* operon during initial investigations (Latour et al., 2013; El Sahili et al., 2015). *qsdR* is located less than 200 bp from the divergently oriented neighboring *qsdA* gene, as is the case for the most genes encoding TetR regulators and their target genes (Ahn et al., 2012; Cuthbertson and Nodwell, 2013). Interestingly, after 3-oxo-C_8_-HSL addition in cultures, the profile of the amounts of *qsdR* transcript showed a pattern of mRNA accumulation similar to that of the *qsdA* and *qsdC* genes (Figure 2). The 5′ end of the mRNA transcribed from the *qsd* operon or the *qsdR* gene was determined by 5′RACE. Individual TSSs were identified for both *qsdA* and *qsdR* RNAs. The *qsdA* and *qsdR* transcription began at a T located 43 and 104 bp upstream of their respective start codons, respectively. In the *qsdR-qsdA* intergenic region, two palindrome motifs with free energy ΔG of -22.3 kJ for IR1 and -49.1 kJ for IR2 were detected and might correspond to putative operator sequences for a potential QsdR binding. Interestingly, the *qsd* cluster bordered by *qsdR* and *qsdC* genes is preceded by a FadR encoding gene located 102 bp downstream of the stop codon of *qsdR*. Besides, a putative binding site for FadR transcriptional factor was predicted upstream of the -35 box of *qsdR* promoter (Supplementary Figure S1). This sequence shares 75% identity with the consensus sequence recognized by FadR of *E. coli* (Bacik et al., 2015).

**FIGURE 3:**
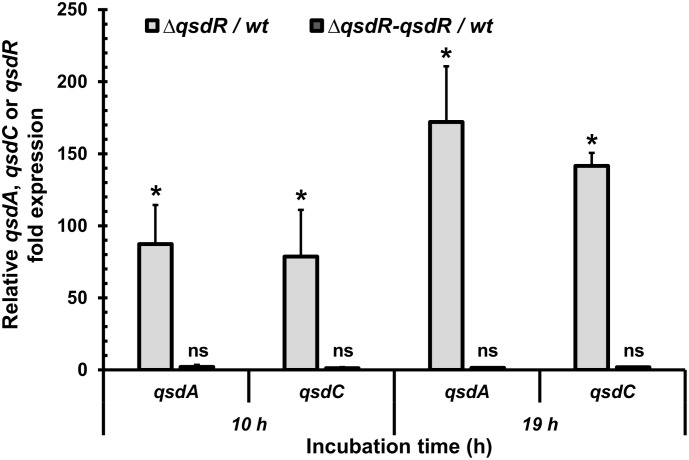
Increased *qsdA* and *qsdC* mRNA levels in a Δ*qsdR* mutant strain. qRT-PCR analysis shows the *qsdA* and *qsdC* mRNA levels in the Δ*qsdR* mutant and in the complementing strain (Δ*qsdR-qsdR*) grown in 7H9 medium at the mid-exponential (10 h) and stationary (19 h) phases. These mRNA levels are relative to the wild type strain. Data shown are mean values obtained from three independent experiments. Statistical analysis was performed by the DataAssist^TM^ software (v3.01) used for calculating relative quantitation of gene expression, based on the comparative *C*_T_ (2^-ΔΔCT^) method. ^∗^*p*-value < 0.05; ns means no significant difference.

To verify the hypothesis that the *qsdR* gene plays a role in regulating the *qsd* operon, a *qsdR* deletion mutant was constructed as described in the Section “Materials and Methods” and the *qsdR*, *qsdA*, and *qsdC* gene expression was analyzed using qRT-PCR and compared with that obtained in the non-induced wild-type strain (Figure 3). As expected, no *qsdR* expression was detected in the Δ*qsdR* deletion mutant. Furthermore, *qsdA* and *qsdC* expression levels were significantly higher than those observed in the wild-type strain, with around 80-fold change after 10 h of incubation and 150-fold change after 19 h of incubation (Figure 3). The *qsdA* and *qsdC* expression levels were restored to wild-type levels in the Δ*qsdR* mutant complemented with the *qsdR* gene on the integrative vector pSET152, corresponding to the Δ*qsdR-qsdR* strain.

**FIGURE 4:**
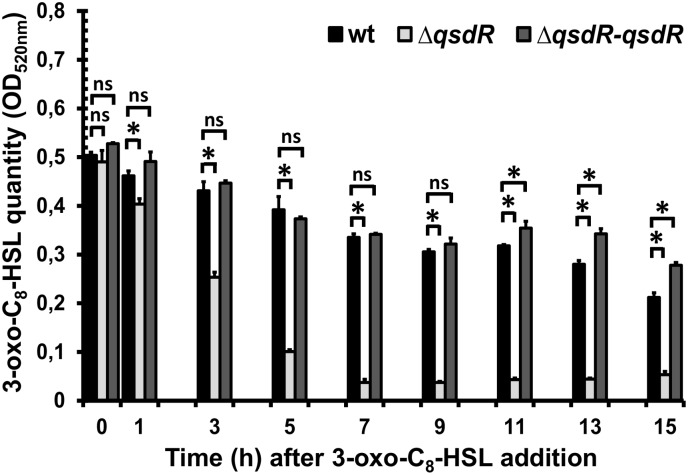
Degradation kinetics of 3-oxo-C_8_-HSL by the *R. erythropolis* R138 wild-type, Δ*qsdR* or the complementing strains. Dosage was performed by a colorimetric method in the culture supernatant after centrifugation. Data are the mean ± standard deviation of three independent experiments. Statistical analyses were performed using non-parametric Mann–Whitney tests (two tailed). For each incubation time, ^∗^ indicates a significant difference in the 3-oxo-C_8_-HSL quantity in the presence of the Δ*qsdR* mutant or the complementing strains (*p*-value < 0.05) relative to that observed in the presence of the wild-type strain. ns mean no significant difference.

In order to analyze the QS signal degrading activity of *R. erythropolis* in the Δ*qsdR* mutant, 3-oxo-C_8_-HSL molecules were added at mid-exponential phase and quantified at different time points along the growth curve by a colorimetric method based on the formation of hydroxamic acids from esters by reaction with hydroxylamine in alkaline solution. In the presence of the wild-type strain, the 3-oxo-C_8_-HSL amount in the medium diminished continuously until reaching 60 and 40% of its initial quantity 9 and 15 h after its addition, respectively (Figure 4). In contrast, the 3-oxo-C_8_-HSL degradation by the Δ*qsdR* mutant was faster than with the wild-type strain. The decrease of 3-oxo-C_8_-HSL reached 50% with the Δ*qsdR* mutant 3 h after the addition of the molecule, whereas with the parental strain it was only of 14%. For the complemented strain, the 3-oxo-C_8_-HSL degradation kinetic followed closely the degradation profile of the wild-type strain (Figure 4).

### Binding of QsdR to the Promoter Regions of the *qsd* Operon Is Prevented by the Binding of the Lactone Ring of QS Signals to the QsdR Effector Domain

**FIGURE 5:**
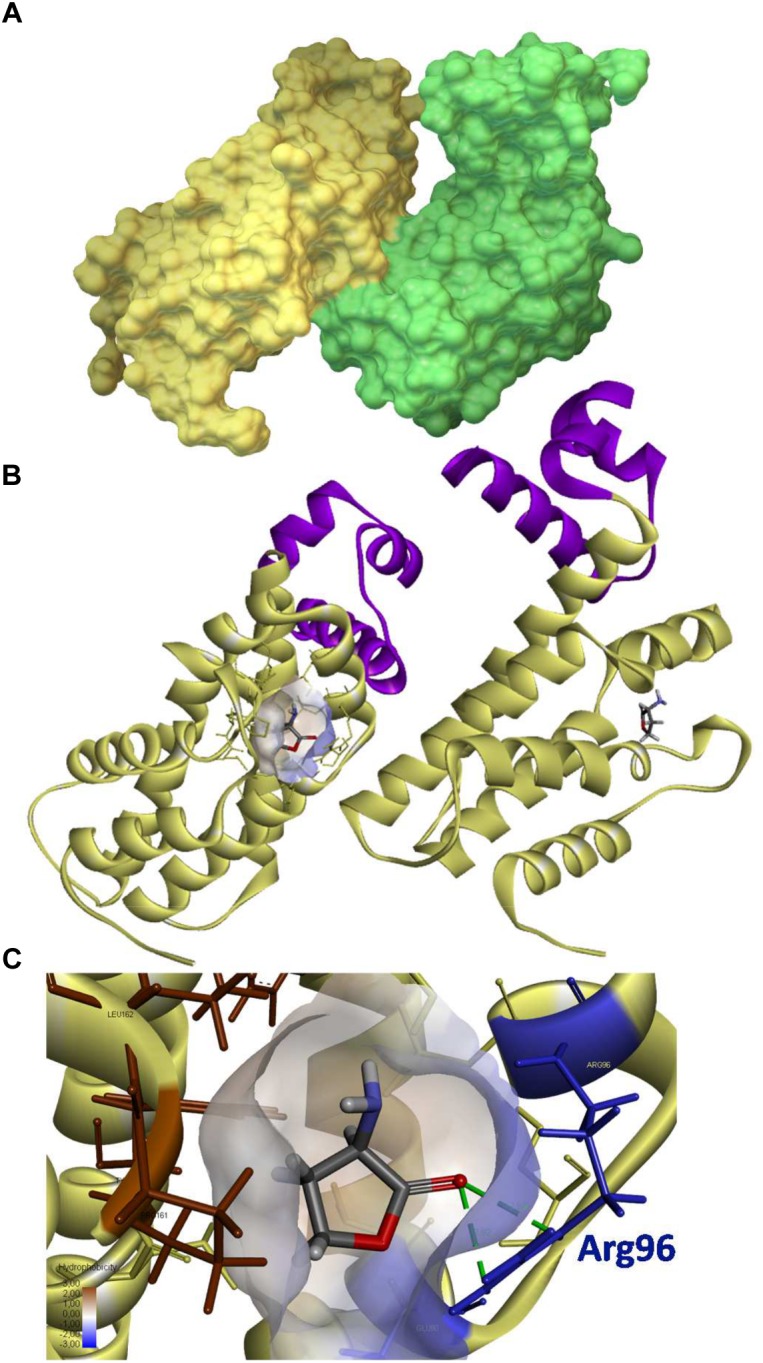
3D structure of QsdR and its potential ligand. The QsdR structure of the R138 strain (RCSB PDB accession number 4ZA6) was elucidated after crystallization and X-Ray diffraction (El Sahili et al., 2015). **(A)** A solvent-accessible surface area representation shows the functional structure of QsdR consisting of two monomers (yellow and green) that interact asymmetrically. **(B)** A secondary structure representation shows that each monomer presents both a DNA-binding domain (N-domain) colored in purple and an effector binding domain (C-domain) indicated in yellow, this last one harboring at the core a narrow half apolar/half polar cavity (drawn only on one of the monomers). **(C)** A close-up of the core cavity and HSL ligand are presented. Molecular docking methodology predicts a binding of the homoserine lactone (HSL), which is also the cyclic moiety of the QS signal, in each of the effector pockets **(B,C)**. The lactone ester bond of the homoserine lactone is oriented toward the hydrophilic part of the pocket (colored in translucent blue) while the rest of the ligand molecule is oriented toward the hydrophobic part (colored in translucent brown). The hydrogen bonds between the oxygen atom of lactone carbonyl and the nitrogen atoms of Arg96 residue are indicated in green dashed line.

**FIGURE 6:**
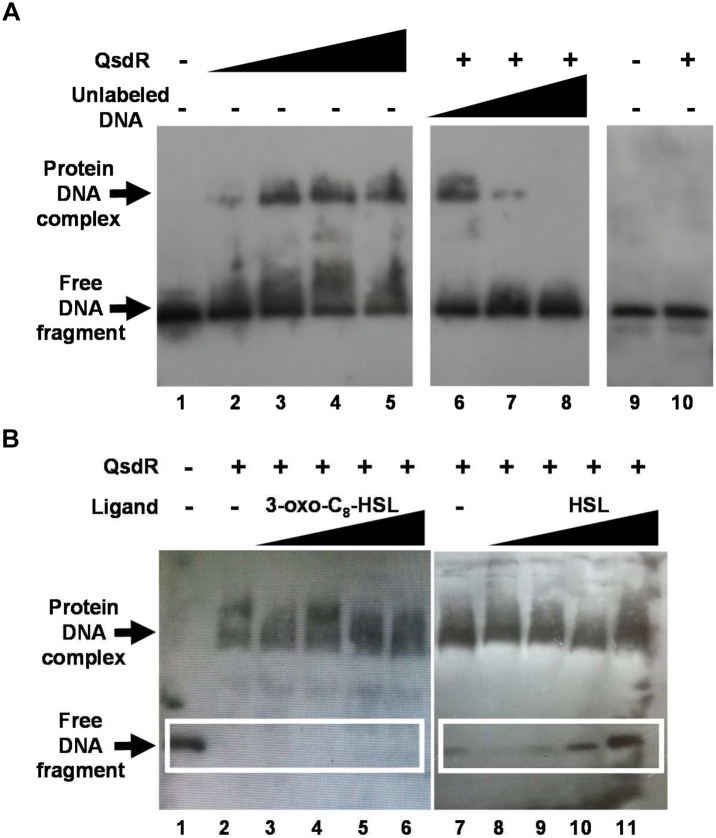
Detection of a QsdR–DNA complex by EMSA using biotin labeled *qsdR qsdA* intergenic DNA fragment. **(A)** Binding of QsdR to 0.4 ng of the *qsdR qsdA* intergenic DNA fragment with increasing amounts of His-tagged QsdR. Lane 1: biotin labeled fragment in the absence of protein (-); lanes 2–5: 0.3, 1, 2, and 3 μg, respectively, of the purified His-tagged QsdR protein; lanes 6–8: 0.4, 0.8, and 4 ng, respectively, of the unlabeled competitor *qsdR qsdA* intergenic DNA fragment with 2 μg of the purified QsdR protein (+); lanes 9–10: EMSA performed with 0.4 ng of a biotin labeled DNA internal fragment of *qsdA* gene, used as negative control, in the absence (-) or in the presence (+) of 2 μg of the purified QsdR protein. **(B)** Effect of ligands on the QsdR binding activity. EMSA was performed under the same conditions as described in panel **(A)** and without (lanes 1, 2, and 7) or with increasing amounts of 3-oxo-C_8_-HSL (lanes 3–6) or HSL (lanes 8–11) molecules by adding a ligand/QsdR molecules ratio increasing from 0.1 to 100 to the reaction binding. The data shown are representative pictures of three independent experiments. Symbols + and - mean the presence or absence of the corresponding molecule, respectively.

The QsdR structure of *R. erythropolis* R138 (RCSB PDB accession number 4ZA6) was elucidated after crystallization and X-Ray diffraction (El Sahili et al., 2015). QsdR encodes a monomer (186 residues) capable of an asymmetric dimerization (Figure 5A). Each monomer presents both a DNA-binding domain (N-domain) and an effector binding domain (C-domain), two traits shared by TetR-like family members (Figure 5B). To determine if QsdR could bind to the DNA intergenic region *qsdR-qsdA*, the purified His-tagged QsdR protein was obtained by cloning *qsdR* ORF into the expression vector pET19 and by purifying the recombinant protein by affinity chromatography. Gel retardation assays were performed with a 5′ end labeled 170 bp PCR product that contained the *qsdR* and *qsdA* promoter regions. Retardation was readily detected when an increased amount of QsdR was added (Figure 6A). Addition of 2- and 10-fold excess of unlabeled promoter containing fragment resulted in a reduction in the proportion of the labeled fragment that was retarded. This competitive binding of unlabeled fragment demonstrated the specificity of the interaction. No retardation was observed with a 5′ end labeled PCR product corresponding to an internal fragment of the *qsdA* gene.

As expected in a TetR-like regulator, a potential binding site appears in the core of each effector domains (Cuthbertson and Nodwell, 2013). This cavity is narrow with both polar and apolar traits, suggesting the binding of small amphiphilic molecules such as small lactone molecules to the QsdR protein (Figure 5B). Conversely, the affinity of QsdR to lactones bearing long aliphatic chain should be limited. In order to identify potential ligand(s) of QsdR, an *in silico* analysis was carried out by molecular docking using the functional structure of QsdR and those of some suspected ligands. Molecules tested included both complete known signaling molecules and the AHL γ-butyrolactone ring, i.e., homoserine lactone (HSL) molecules. Among those molecules, only the HSL showed a docking in the effector binding site of each QsdR monomers (Figures 5B,C). The calculated affinity value (-17.6 kJ/mol) was sufficient to support a strong interaction with HSL and was identical for each QsdR binding pockets. QsdR–HSL interaction should be favored by (i) the close correspondence between the size of the ligand and that of the binding site, (ii) the presence of two hydrogen bonds (2.92 and 2.93 Å length) between the lactone carbonyl oxygen and the Arg96 residue, and (iii) the amphiphilic character of the effector cavity. The latter should involve the Glu90 and Arg96 polar residues and the Phe106, Leu158, Tyr159, Pro161, and Leu162 apolar residues (Figure 5C).

Based on the results of molecular docking, HSL and 3-oxo-C_8_-HSL interaction was investigated by gel retardation assays to evaluate their ability to affect the DNA binding activity of QsdR. Increasing amounts of HSL or 3-oxo-C_8_-HSL were used in a 10-fold dilution series in binding reactions of the labeled fragment with a constant concentration of QsdR. Addition of increased amounts of HSL induced a proportional increase in free DNA, indicating a reduction of DNA-QsdR complex formation. By contrast, 3-oxo-C_8_-HSL failed to inhibit the DNA binding activity of QsdR (Figure 6B). These results are fully consistent with molecular docking studies.

### A Transcriptional Fusion Approach Allows Proposal of a Model for the Regulation of the *qsd* Operon

**FIGURE 7:**
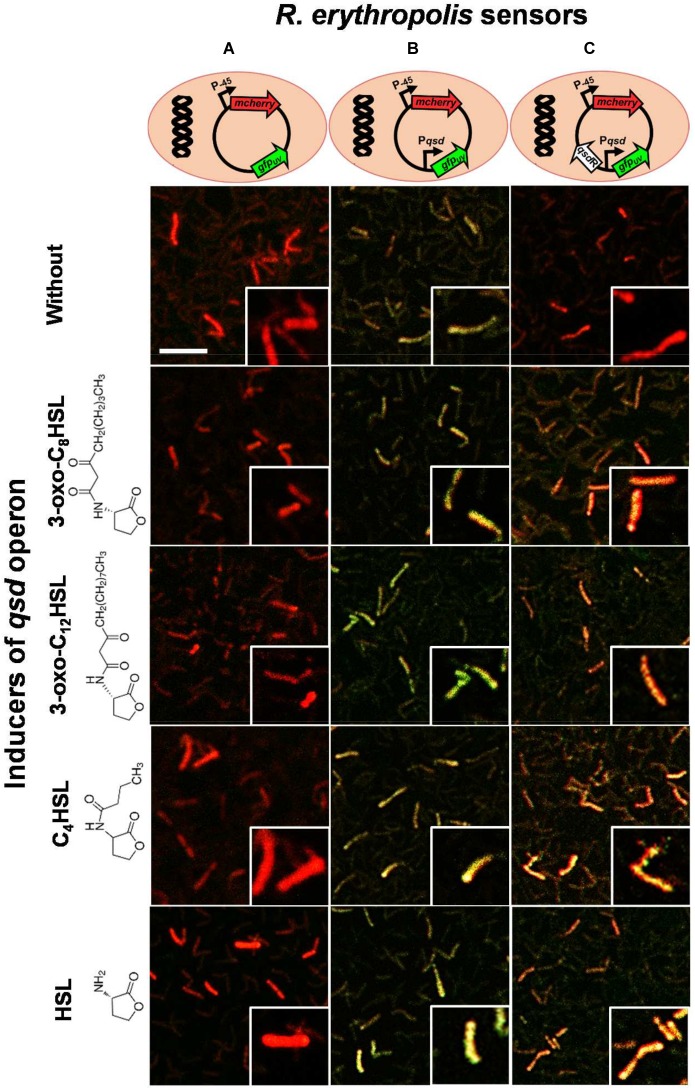
Confocal microscopy analysis of the transcriptional activity of the *qsd* operon. Three dual-colored *R. erythropolis* R138 sensors were constructed using the pEPR1 vector containing a *green fluorescent protein* gene as reporter gene and a red fluorescent protein *mcherry* gene as a cell tag. These strains carry **(A)** the *gfp* gene without promoter (as a control), **(B)** the *gfp* gene under control of the *qsd* promoter, and **(C)** the *gfp* gene under control of the *qsd* promoter and QsdR regulator (mimicking the regulation system of *qsd* operon expression). Rhodococcal cells were cultured in hexanoate minimal medium and harvested every 2 h after the addition of one micromolar of 3-oxo-C_8_-HSL, 3-oxo-C_12_-HSL, C_4_-HSL, or HSL. Cultures in hexanoate without inducer were used as control. Map of each designed vector are schematized at the top of the figure and the structure of inducers with the common γ-lactone ring is drawn on the left of the figure. Fluorescence images were combined in the green and red channels (see section “Materials and Methods”), the co-expression of the two fluorescent proteins causing a yellow-greenish to amber fluorescence depending on the intensity of *gfp* expression. A close-up of some cells is proposed in a lower right window. Scale bar represents 10 μm.

In order to go further into the validation of the interaction between QsdR and QS signals, we constructed a vector pEPR1-*qsdR*-P*qsd*::*gfp-mCherry* that mimics the regulation system of the *qsd* operon. Firstly, a *mCherry* cassette under constitutive promoter was introduced into the pEPR1 vector to tag bacteria constitutively in red fluorescence (Figure 7A). Secondly, the promoter probe vector pEPR1-*mCherry* was modified by introducing the *qsd* promoter upstream of the *gfp* ORF to make a P*qsd*::*gfp* transcriptional fusion and investigate *qsd* promoter activity. Confocal microscopy analysis of R138 pEPR1-P*qsd*::*gfp-mCherry* cultures without inducer (AHL) molecules revealed the presence of yellow-greenish tagged bacteria, resulting from the expression of a mixture of red and green fluorescent proteins (Figure 7B). This demonstrates a constitutive expression of *gfp* under the control of *qsd* promoter. Thirdly, the introduction of the *qsdR* gene into the pEPR1-P*qsd*::*gfp-mCherry* vector led to the observation by confocal microscopy of *mCherry* producing R138 pEPR1-*qsdR*-P*qsd*::*gfp-mCherry* bacteria in the absence of inducer molecules (Figure 7C). The absence of *gfp* expressing bacteria demonstrates the role of QsdR as a repressor of the *qsd* promoter activity. Addition of 3-oxo-C_8_-HSL molecules in R138 pEPR1-*qsdR*-P*qsd*::*gfp-mCherry* cultures induced the appearance of yellow tagged bacteria 2 h after the onset of the experiment (Figure 7C). Analysis by confocal microscopy of R138 pEPR1-*qsdR*-P*qsd*::*gfp-mCherry* cultivated with different inducer concentrations (from 10 nM to 1 mM) led to the determination of a threshold 3-oxo-C_8_-HSL concentration for *qsd* promoter induction. This was estimated to 1 μM. Interestingly, identical results were obtained when 3-oxo-C_12_-HSL or C_4_-HSL were used as inducer (Figure 7), or when identical concentrations of HSL were used in place of the signaling molecules (Figure 7). These results confirmed the key role of the lactone ring structure in this regulatory mechanism.

## Discussion

The existence of communication mechanisms within microbial populations should imply the concomitant existence of signal sensing and disrupting systems in competing environmental populations belonging to common ecological niches (Reimmann et al., 2002; D’Angelo-Picard et al., 2005). Knowledge of such systems should permit better understanding of the ecological roles dedicated to QQ and their various potential applications (Njoroge and Sperandio, 2009; Grandclément et al., 2016; Zhang and Li, 2016). However, due to the lack of screening systems, QQ remains as a mechanism under-explored among the promising *Actinobacteria* as pointed out by Polkade et al. (2016). Members of the *R. erythropolis* species are characterized by their remarkable metabolic versatility with specific enzymatic abilities to degrade recalcitrant and xenobiotic compounds (van der Geize and Dijkhuizen, 2004; de Carvalho and da Fonseca, 2005; Larkin et al., 2005; Martínková et al., 2009). This metabolic capacity is notably illustrated in strain R138 by the *qsd* pathway involved in its biocontrol activity against soft-rot phytopathogens (Barbey et al., 2013). This pathway strongly implicates the lactonase QsdA (Uroz et al., 2008; Barbey et al., 2012). Even though the 3-oxo-C_8_-HSL appears as one of the QsdA’s best known substrate, a wide range of AHLs including C_4_-HSL and 3-oxo-C_12_-HSL may be hydrolyzed by this intracellular lactonase enzyme (Afriat et al., 2006; Uroz et al., 2008; Fetzner, 2015). This broad substrate specificity is confirmed *in vivo* by the rhodococcal control of potato soft-rot, and by the rhodococcal suppression of tuber necrosis due to *P. aeruginosa* PA14 without bacterial population diminution (Figure 1). Since the virulence of the PA14 strain was weakly affected in the presence of the *R. erythropolis* mutant strain defective in the production of the lactonase QsdA, the biocontrol activity against *P. aeruginosa* may be explained by the degradation of 3-oxo-C_12_-HSL and C_4_-HSL signaling molecules. These AHL signals are known to regulate the production of *Pseudomonas* virulence factors altering both plant and animal host cells, like phospholipase C and exotoxin A (Rahme et al., 2000; Reimmann et al., 2002).

### Rhodococcal Quorum-Quenching Activity Is Regulated by a TetR-Like Repressor

The QQ QsdA enzyme is encoded by the *qsd* operon, the expression of which has been shown *in planta* to be induced by AHL when a quorum pathogen density is reached (Barbey et al., 2013). This induction by AHLs was confirmed in the present work by analyzing the *qsd* operon expression by qRT-PCR and by the transcriptional fusion approaches. The latter led to the establishment of a threshold concentration of 1 μM for induction by AHLs. This concentration was observed for all studied AHLs. This value might have ecological significance since it corresponds to previous measurements recorded in soft-rotting tuber colonized by the *P. atrosepticum* CFBP6276 strain (2.5 μM) (Barbey et al., 2013) or *P. atrosepticum* SCRI1043 strain (290 μM) (Liu et al., 2008).

**FIGURE 8:**
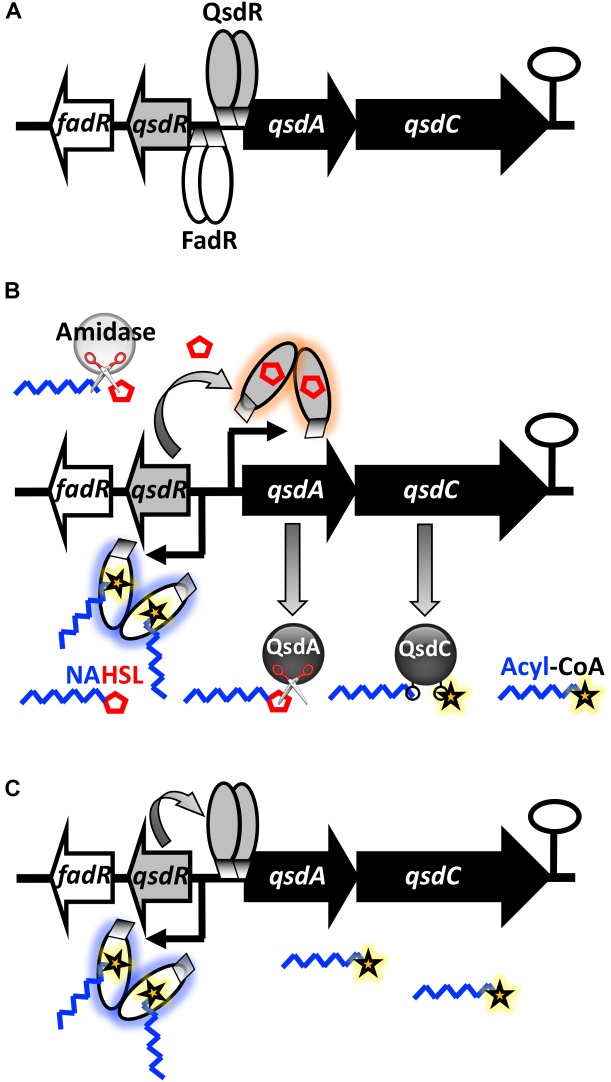
Proposed model for the regulation of the *qsd* operon expression by quorum-sensing signals in *R. erythropolis*. **(A)** In the absence of an inducer, the TetR-like QsdR regulator forms a dimer that binds to the promoter region and directly represses *qsdA* and *qsdC* expression, switching off the pathway. **(B)** When *N-*acyl-L-homoserine lactones (AHLs) are present in cellular environment, some of them are cleaved by the previously characterized extra-operonic gene encoded AHL amidase (Latour et al., 2013) which hydrolyses the amide bond of these molecules (symbolized by scissors). The γ-butyrolactone ring moiety (i.e., homoserine lactone) binds to QsdR, thereby changing the conformation, such that QsdR cannot bind to the promoter region, inducing derepression of the *qsd* cluster gene expression. This leads to the production of QsdA and QsdC enzymes, responsible for the lactone ester bond hydrolysis and activation (coenzyme A ligation) of the aliphatic part of signaling molecules, respectively. **(C)** When AHL molecules are fully hydrolyzed, the disappearance of HSL rings restores the binding between QsdR and the DNA promoter sequence leading to the lock of *qsd* operon expression by the QsdR repressor. The concomitant increasing in the intracellular amount of acyls-CoA molecules facilitates this phenomenon, since these molecules bind to the effector domain of the FadR regulator, causing a conformational change in the dimeric protein and the production of the QsdR repressor. This step is proposed according to *in silico* analysis of the genome of *R. erythropolis* strain R138 and the recent and well-documented bibliography related to the functioning of FadR regulator (Bacik et al., 2015; My et al., 2015). (Black curved arrows and hairpin represents the initiation of gene transcription and a putative transcription terminator, respectively.)

Previous observations added to *in silico* DNA analysis confirms the existence of a regulator for the *qsd* operon expression named QsdR (Latour et al., 2013; El Sahili et al., 2015). QsdR belongs to the TetR transcriptional regulator family, which represents a large and important family of one-component signal transduction systems (Cuthbertson and Nodwell, 2013). The disruption of *qsdR* resulted in high level expression of the *qsd* operon, preventing the repression of its expression. This lifting of repression mechanism was phenotypically observed with the enhancement of the AHL degrading activity in the Δ*qsdR* strain, compared to the wild-type strain. Furthermore, EMSA experiments demonstrated a physical binding of QsdR to the *qsd* promoter region. All of these results support the hypothesis that QsdR acts as a negative regulator for the *qsd* operon expression, as reported for most TetR-like regulators (Ramos et al., 2005; Cuthbertson and Nodwell, 2013). A simplified model of the *qsd* operon repression is illustrated in Figure 8A: the absence of QsdA and QsdC products results from repression of transcription by QsdR binding to the *qsd* promoter region. TetR family regulators are known to regulate the expression of efflux pumps and transporters involved in antibiotic resistance and tolerance to toxic chemicals (Ramos et al., 2005; Reichheld et al., 2009). A large majority of them also control other functions: carbon, nitrogen and co-factor metabolism as well as cell division and cell-to-cell signaling (Ramos et al., 2005; Cuthbertson and Nodwell, 2013). Interestingly, another TetR-like regulator implicated in lactone metabolism was described in *R. erythropolis*: this is the LplR repressor involved in the regulation of the L-pantoyl lactone dehydrogenase encoding gene expression essential for the vitamin B5 synthesis (Si et al., 2012). Besides, a TetR based regulation of a lactonase encoding gene expression was reported in *A. tumefaciens*: the transcriptional repressor BclR regulates the expression of *blcABC* operon which codes for the BclC lactonase (Khan and Farrand, 2009; Lang and Faure, 2014). However, *bclC* expression was demonstrated to be induced by a byproduct of γ-butyrolactone metabolism, succinate semialdehyde, but not by AHL signals or its byproducts.

### Quorum-Sensing Signals Indirectly Activate Quorum-Quenching Activity

According to qRT-PCR and transcriptional fusion assays, addition of the QS signal induced an increase in *qsd* operon expression and in *qsd* promoter activity, respectively, demonstrating a role of these inducers in derepression of gene expression. In the well-characterized TetR family regulators, this derepression is usually operated by the direct binding of small-molecule ligands to the signal-receiving domain of TetR, that causes a conformational change preventing its specific binding at the promoters of target genes (Reichheld et al., 2009; Cuthbertson and Nodwell, 2013). Generally, ligands involved in metabolism regulation correspond to the substrate intended to be degraded or a catabolic intermediate of the target gene product (Cuthbertson and Nodwell, 2013). A docking approach predicted the binding of the AHL γ-butyrolactone ring (i.e., HSL) molecules to the core of each QsdR effector domain. These results were experimentally directly supported by a decrease in DNA-protein complex formation only in the presence of HSL in EMSA experiments and indirectly by confocal microscopy GFP fluorescence analysis (Figure 7). Thus, the induction of the *qsd* operon expression by QS signals seems to be operated by HSL, especially since a same threshold induction value (1 μM) has been recorded for HSL and AHLs (Figure 7). However, we cannot exclude the existence in the environment of other ligands whose structure should be close to that of HSL, nor the involvement of the *qsd* pathway in the degradation of other QS signals such as the 2,3-disubstituted γ-butyrolactones used by some *Streptomyces* (Takano, 2006) and *Rhodococcus* (Latour et al., 2013; Ceniceros et al., 2017a,b) species. In this case, the QQ pathway of *R. erythropolis* would be also involved in the turn-over of its QS signals and therefore the regulation of its own communication.

The HSL-QsdR binding requires the implication of an extra-operonic gene encoded AHL amidase to release the lactone ring from the signaling molecules. This hydrolysis should generate two byproducts, a fatty acid and one molecule of free HSL, the latter serving as an inducer of their own catabolism (Figure 8B). This AHL cleavage hypothesis is supported by (i) the AHL amide bond cleavage activity previously detected in *R. erythropolis* (Uroz et al., 2005), (ii) the fact that the deletion of the *qsdA* gene did not completely abolish the biocontrol activity of the strain R138 (Figure 1) implying the complementary activity of another AHLase, and above all (iii) the characterization of an AHL amidase (NCBI reference WP_019744590.1) in the R138 strain proteome obtained after bacterial growth with 3-oxo-C_8_-HSL as a sole C source (Latour et al., 2013). In this case, the question of the substrates diversity of this amidase arises, since it will determine the range of AHLs that can be degraded. Most AHL amidases display a preference for long-chain AHLs with or without a substituent at C_3_ of the acyl chain (Fetzner, 2015). However, AiiC produced by *Anabaena* sp. PCC7120 (Romero et al., 2010) is involved in the hydrolysis of a wide range of AHLs including C_4_-HSL, the signal of the *rhl* system of *P. aeruginosa*.

The regulation of the *qsd* operon expression probably involves autoregulation systems. The expression of QsdR should be influenced by fatty acid metabolism due to the presence of both a FadR encoding gene located immediately downstream of *qsdR* and a FadR binding site predicted upstream of the -35 box of the *qsdR* promoter (Supplementary Figure S1). FadR is a fatty acid degradation transcriptional regulator controlling fatty acid metabolism in response to intracellular concentrations of acyl-CoA esters (DiRusso et al., 1992; My et al., 2015). In the *E. coli* model, FadR repression of *fad* genes expression is due to its binding to the promoter sites. This repression is lifted by interaction between FadR and long-chain acyls-CoA that have accumulated inside the cell (Bacik et al., 2015; My et al., 2015). In Gram-positive bacteria, FadR also regulates several operons required for fatty acid degradation and recognizes fatty acyls-CoA as effectors (Matsuoka et al., 2007). The existence and location of such regulator was first reported in the *R. erythropolis* species by Uroz et al. (2008) and echoes the presence of the QsdC (syn. FadD) fatty acyls-CoA ligase encoded by the *qsd* operon. Indeed, in *E. coli*, acyl-CoA esters resulting from FadD activity on fatty acids bind to FadR, repressing the expression of catabolic genes (Pech-Canul et al., 2011). This suggests a control of *qsdR* expression by FadR and consequently a ligand role for the acyl part of QS signals after ligation with coenzyme A (Figures 8A,B). In this hypothesis, the *qsd* operon expression might be locked in response to the hydrolysis of all γ-lactone rings coupled with the presence of a large amount of acyl-CoA esters resulting from QsdA and QsdC activities on signaling molecules (Figure 8C). This hypothesis might explain the decrease in the *qsdR* expression following the 3-oxo-C_8_-HSL molecules consumption by *Rhodococcus* (Figure 2). Significant work remains to be done to understand the possible role of FadR in this regulatory mechanism.

## Conclusion

This work provides evidence for AHL-based regulation of QQ enzymes in *Rhodococcus* which appear to cause a bacterial communication interference. This system seems well adapted to the detection and the degradation of a wide range substrates, since it appears based on detection of the common structure (the HSL ring) of different AHLs. Since signal specificity of AHLs is determined by the length and the substitution of the acyl chain (Brader et al., 2005; Welch et al., 2005; Crépin et al., 2012a), such a regulatory mechanism appears to have a global metabolic action toward the AHL family rather than targeting the communication of a particular population. This asset may confer a selective advantage to rhodococci to successfully compete for resource uptakes and colonization in the same ecological niche as many AHL-producing bacteria (e.g., biofilm and rhizosphere) (Cha et al., 1998; D’Angelo-Picard et al., 2005). This hypothesis is also supported by the fact that 3-oxo substituted-HSL molecules have been demonstrated to be bactericidal in Gram-positive bacteria (Kaufmann et al., 2005). Future studies should elucidate and integrate the role of amidase in the QsdR based regulation system.

## Author Contributions

CB, AC, J-FB, and XL conceived and designed the experiments. CB, AC, and OM performed the experiments. CB, AC, and XL analyzed the data and wrote the paper. AM, YK-G, MG, AB-C, VG, and MF participated to technical and scientific discussion.

## Conflict of Interest Statement

AB-C and VG were employed by an Agricultural Technical Institute, the French Federation of Seed Potato Growers. The remaining authors declare that the research was conducted in the absence of any commercial or financial relationships that could be construed as a potential conflict of interest.
