# Supplementary material for: A Rhodococcal Transcriptional Regulatory Mechanism Detects the Common Lactone Ring of AHL Quorum-Sensing Signals and Triggers the Quorum-Quenching Response
Source: Front Microbiol. 2018 Nov 19;9:2800. doi: 10.3389/fmicb.2018.02800 (PMC6262395; doi:10.3389/fmicb.2018.02800)
Supplement: Supplementary file 1 [file Table_1.DOCX]

**TABLE S1. Bacterial strains and plasmids**

| **Strain or plasmid** | **Relevant characteristic(s)** | **Source or reference** |
| --- | --- | --- |
| ***Escherichia coli*** |  |  |
| S17-1 | *recA pro hsdR* RP4-2-Tc::Mu-Km::Tn*7* | Simon et al., 1983 |
| DH5α | Host for cloning; *supE44* Δ*lacU169* (Φ80*lacZ*ΔM15) *hsdR17 recA1 endA1 gyrA96 thi-1 relA1* | Lab collection |
| BL21DE3 | Host for production of recombinant protein; F^-^*ompT* *hsdSB*(*rB^-^mB^-^*)*gal dcm* | Novagen |
| DH5α (pUC19) | Strain DH5α carrying pUC19; Ap^R^ | Barbey et al., 2013 |
| DH5α (pUC19-*qsdA*) | QsdA-producing DH5α ; Ap^R^ | Barbey et al., 2013 |
| ***Rhodococcus erythropolis*** | | |
| R138 | AHL degrading isolate obtained from hydroponic culture of potato plants | Cirou et al., 2007 |
| R138 Δ*qsdA* | R138 with a 813 bp fragment deleted from the *qsdA* gene | Barbey et al., 2013 |
| R138 Δ*qsdR* | R138 with a 489 bp fragment deleted from *qsdR* gene | This study |
| R138 Δ*qsdR+qsdR* | R138Δ*qsdR* transformed by pSET152-*qsdR* | This study |
| R138 pEPR1-*mCherry* | R138 transformed by pEPR1-*mCherry* | This study |
| R138 pEPR1 -P*qsdA*::*gfp*-*mCherry* | R138 transformed by pEPR1-P*qsdA*::*gfp*-*mCherry* | This study |
| R138 pEPR1-*qsdR*-P*qsdA*::*gfp*-*mCherry* | R138 transformed by pEPR1-*qsdR*-P*qsdA*::*gfp*-*mCherry* | This study |
| **Bacterial pathogens** |  |  |
| *Pectobacterium atrosepticum* 6276 | Potato soft-rot pathogen; AHL producer | Smadja et al., 2004b |
| *Pseudomonas aeruginosa* PA14 | Human clinical isolate; AHL producer | Rahme et al., 1997 |
| **Plasmids** |  |  |
| pAKE604 | Conjugative suicide vector for *qsdR* gene deletion; Km^R^ | El-Sayed et al., 2001 |
| pAKE604 Δ*qsdR* | pAKE604 containing the *qsdR* upstream and downstream regions; Km^R^ | This study |
| pSET152 | Integrative vector containing the bacteriophage ϕ-C31 attachment site; Am^R^ | Swain et al.,2012 |
| pSET152-*qsdR* | pSET152 containing the *qsdR* gene with its promoter | This study |
| pET19-*qsdR* | pET19 expression vector containing the *qsdR* ORF | This study |
| pEPR1 | Vector for testing *in vivo* promoter activities in *R. erythropolis*; Km^R^ | Knoppová et al., 2007 |
| pEPR1-*mCherry* | pEPR1 vector containing a *mCherry* cassette under constitutive promoter; Km^R^ | This study |
| pEPR1- P*qsd*::*gfp*-*mCherry* | pEPR1 vector containing a transcriptional fusion with the *gfp* gene under the control of the *qsd* promoter; Km^R^ | This study |
| pEPR1-*qsdR*-P*qsdA*::*gfp*-*mCherry* | pEPR1 vector containing the *qsdR* gene, a transcriptional fusion with the *gfp* gene under the control of the *qsd* promoter and a *mCherry* cassette under constitutive promoter; Km^R^ | This study |
| pPSV35-mCherry | pPSV35 vector containing a mCherry cassette under constitutive promoter ; Gm^R^ | Lab collection |
| pUC19 | Cloning vector for *E. coli*; Ap^R^ | Barbey et al. 2013 |
| pUC19-*qsdA* | pUC19 with a 1376 bp PCR fragment containing the *qsdA* gene; Ap^R^ | Barbey et al. 2013 |

Km^R^, Gm^R^, Am^R^ and Ap^R^ indicate resistance to kanamycin, gentamycin, apramycin, and ampicillin, respectively. AHL, *N*-acyl homoserine lactone.
