# Supplementary material for: A Rhodococcal Transcriptional Regulatory Mechanism Detects the Common Lactone Ring of AHL Quorum-Sensing Signals and Triggers the Quorum-Quenching Response
Source: Front Microbiol. 2018 Nov 19;9:2800. doi: 10.3389/fmicb.2018.02800 (PMC6262395; doi:10.3389/fmicb.2018.02800)
Supplement: Supplementary file 2 [file Table_2.DOCX]

**TABLE S2. Primers used for cloning, qRT-PCR, RACE and EMSA assays**

| **Primer name** | | **Sequence*** | **Reference** |
| --- | --- | --- | --- |
| ***qsdR* deletion** | |  |  |
| qsdR_up_fw | | 5’ TAATAA*GAATTC*AGAACATCGAGACCGAAACG 3’ | This study |
| qsdR_up_rv | | 5’ TAATAA*AAGCTT*TAGGCATCGGGACATTCTCT 3’ | This study |
| qsdR_do_fw | | 5’ TAATAA*AAGCTT*AAAAGGCGGAGATCGAGACG 3’ | This study |
| qsdR_do_rv | | 5’ TAATAA*CCCGGG*CTCTCTTAGGCGTCGAGCAC 3’ | This study |
| **Complementing construction for the *qsdR* mutation** | | | |
| pSET152_qsdR_F | | 5’ TAATAA*GAATTC*CTCATCGATCGAACCCCTGA 3’ | This study |
| pSET152_qsdR_R | | 5’ TAATAA*TCTAGA*GCGAAGTGTGAATCCTGTG 3’ | This study |
| **qRT-PCR assays** | |  |  |
| RecA-F | | 5’ GGCACCTGATCGGGACAA 3’ | This study |
| RecA-R | | 5’ CCGAACCCTTGCCGAAGT 3’ | This study |
| QsdA-F  QsdA-R | | 5’ ACGAGCATGTCTTCGTTCTG 3’  5’ GGATCGACGATCGTGCTGAT 3’ | This study  This study |
| QsdR-F | | 5’ ACCATCGGTTCGCGTTTG 3’ | This study |
| QsdR-R | | 5’ TCATGCGGTCGGTGGAAT 3’ | This study |
| QsdC-F | | 5’ GGCGACGGTTGGTTTTACA 3’ | This study |
| QsdC-R | | 5’ TTTGAAGCCCGAGGCATTT 3’ | This study |
| **Mapping of *qsdA* and *qsdR* transcriptional start sites** | | | |
| qsdA-RACE1  qsdA-RACE2  qsdA-RACE3  qsdR-RACE1  qsdR-RACE2  qsdR-RACE3  Abridged Anchor Primer  Abridged Universal Amplification Primer | | 5’ GCCTCACTCGAGAACCAGTC 3’  5’ TAGTCCAGGTCGGTCGAATC 3’  5’ AGACATGCTCGTGCATCAAC 3’  5’ GAAAGCGTGATCGTCAGTTG 3’  5’ ACCATCGGTTCGCGTTTG 3’  5’ TGCGAAGTGTGAATCCTGTG 3’  5’ GGCCACGCGTCGACTAGTACGGGIIGGGIIGGGIIG 3’  5’ GGCCACGCGTCGACTAGTAC 3’ | This study  This study  This study  This study  This study  This study  Invitrogen  Invitrogen |
| **Construction of the pET19-qsdR vector** | | | |
| pET19-qsdR-F  pET19-qsdR-R | | 5’ TAATAA*CATATG*CCTACCGACCTCGAACG 3’  5’ TAATAA*GGATCC*CGTCAGTTACGGGTGACGCCGA 3’ | This study  This study |
| **DNA fragments used in EMSA** | | | |
| qsdR-qsdA-EMSA F  qsdR-qsdA-EMSA R  qsdA-qsdA-EMSA F  qsdA-qsdA-EMSA R | | 5’ GGTAGGCATCGGGACATTCT 3’  5’ CTCATCGATCGAACCCCTGA 3’  5’ CATTCCTCAAGTGCGCCATC 3’  5’ TCAGATCTGCACCTTCCTCG 3’ | This study  This study  This study  This study |
| **Construction of transcriptional fusions** | | |  |
| mCherry_F | 5’ AATACACTGCCCGTTTCAGG 3’ | | This study |
| mCherry_R | 5’ TAATAA*GCTAGC*GAATTCTTATTTGTAGAGTTCATCCA 3’ | | This study |
| Pqsd_F | 5' CCAATGCATGGTAGGCATCGGGACATTCT 3' | | This study |
| Pqsd_R | 5' CGCGGATCCCGATCGAACCCCTGACTGT 3' | | This study |
| qsdR-Pqsd_F | 5’ TAATAA*ATGCAT*TGCGAAGTGTGAATCCTGTG 3’ | | This study |
| qsdR-Pqsd_R | 5’ TAATAA*GGATCC*CGATCGAACCCCTGACTGT 3’ | | This study |

*All primers used in this study were synthesized by Eurogentec and are based on *R. erythropolis* R138 genome sequence (NCBI accession no. NZ_CM002793.1); restriction site is indicated in italics; the abridged anchor primer contains deoxyinosine residues (I) in the poly (dG) portion.
