# Supplementary material for: A Rhodococcal Transcriptional Regulatory Mechanism Detects the Common Lactone Ring of AHL Quorum-Sensing Signals and Triggers the Quorum-Quenching Response
Source: Front Microbiol. 2018 Nov 19;9:2800. doi: 10.3389/fmicb.2018.02800 (PMC6262395; doi:10.3389/fmicb.2018.02800)
Supplement: Supplementary file 3 [file Data_Sheet_1.PDF]

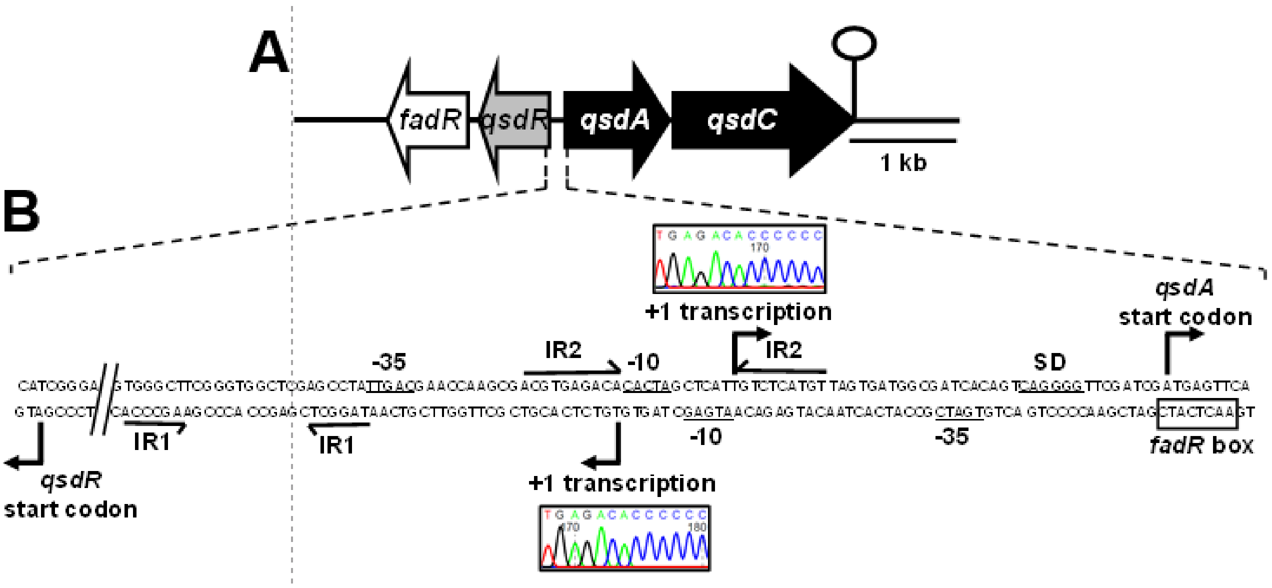

Figure S1. (A) Scaled ORF organization and (B) *in silico* analysis of the intergenic sequence of the *qsd* cluster. In the intergenic sequence, the start codon of *qsdR* and *qsdA* is designated by a curved arrow. Bold convergent arrows indicate inverted repeats, IR1 and IR2, putative operator sequences for transcriptional regulator binding. The transcriptional start site (+1) of the *qsdR* and *qsdA* genes were identified by 5′-RACE PCR, as shown by the relevant part of the chromatogram displaying the corresponding PCR product sequence. The deduced -35 and -10 boxes and the Shine-Dalgarno (SD) sequence are underlined. The web-based software BPROM predicted a putative FadR binding site which is framed. The *qsd* cluster is preceded by a putative FadR encoding gene located 102 bp downstream of the stop codon of *qsdR*. Vertical double bars indicate a 49 bp deletion in the *qsdR*-*qsdA* intergenic sequence.
